# Supplementary material for: Development and validation of a 30-day mortality index based on pre-existing medical administrative data from 13,323 COVID-19 patients: The Veterans Health Administration COVID-19 (VACO) Index
Source: PLoS One. 2020 Nov 11;15(11):e0241825. doi: 10.1371/journal.pone.0241825 (PMC7657526; doi:10.1371/journal.pone.0241825)
Supplement: S1 Table — (DOCX) [file pone.0241825.s002.docx]

**S1 Table. Charlson Comorbidity Index Determination from ICD-10 Diagnosis Codes**

| **Comorbidity** | **ICD-10 Diagnosis Codes** | **Weight** |
| --- | --- | --- |
| AIDS (acquired immunodeficiency syndrome) | B20.x–B22.x, B24.x | 6 |
| Cancer | C00.x–C26.x, C30.x–C34.x, C37.x–C41.x, C43.x, C45.x–C58.x, C60.x–C76.x, C81.x–C85.x, C88.x, C90.x–C97.x | 2 |
| Cancer, metastatic | C77.x–C80.x | 6 |
| Cerebrovascular disease | G45.x, G46.x, H34.0, I60.x–I69.x | 1 |
| Chronic pulmonary disease | I27.8, I27.9, J40.x–J47.x, J60.x–J67.x, J68.4, J70.1, J70.3 | 1 |
| Congestive heart failure (CHF) | I09.9, I11.0, I13.0, I13.2, I25.5, I42.0, I42.5–I42.9, I43.x, I50.x, P29.0 | 1 |
| Dementia | F00.x–F03.x, F05.1, G30.x, G31.1 | 1 |
| Diabetes | E10.0, E10.1, E10.6, E10.8, E10.9, E11.0, E11.1, E11.6, E11.8, E11.9, E12.0, E12.1, E12.6, E12.8, E12.9, E13.0, E13.1, E13.6, E13.8, E13.9, E14.0, E14.1, E14.6, E14.8, E14.9 | 1 |
| Diabetes with complications | E10.2–E10.5, E10.7, E11.2–E11.5, E11.7, E12.2–E12.5, E12.7, E13.2–E13.5, E13.7, E14.2–E14.5, E14.7 | 2 |
| Liver disease, mild | B18.x, K70.0–K70.3, K70.9, K71.3–K71.5, K71.7, K73.x, K74.x, K76.0, K76.2–K76.4, K76.8, K76.9, Z94.4 | 1 |
| Liver disease, moderate or severe | I85.0, I85.9, I86.4, I98.2, K70.4, K71.1, K72.1, K72.9, K76.5, K76.6, K76.7 | 3 |
| Myocardial infarction (MI) | I21.x, I22.x, I25.2, I09.9, I11.0, I13.0, I13.2, I25.5, I42.0, I42.5–I42.9, I43.x, I50.x, P29.0 | 1 |
| Peptic ulcer disease (PUD) | K25.x–K28.x | 1 |
| Peripheral vascular disease (PVD) | I70.x, I71.x, I73.1, I73.8, I73.9, I77.1, I79.0, I79.2, K55.1, K55.8, K55.9, Z95.8, Z95.9 | 1 |
| Plegia | G04.1, G11.4, G80.1, G80.2, G81.x, G82.x, G83.0–G83.4, G83.9 | 2 |
| Renal disease | I12.0, I13.1, N03.2–N03.7, N05.2–N05.7, N18.x, N19.x, N25.0, Z49.0–Z49.2, Z94.0, Z99.2 | 2 |
| Rheumatic disease | M05.x, M06.x, M31.5, M32.x–M34.x, M35.1, M35.3, M36.0 | 1 |

Index calculated by summing comorbidity weights. Three comorbidity pairs are mutually exclusive: Cancer or Cancer, metastatic; Diabetes or Diabetes with complications; and Liver disease, mild or Liver disease, moderate or severe – only use the higher weight, not both.

Adapted from Quan *et al*., Coding algorithms for defining comorbidities in ICD-9 and ICD-10 administrative data. *Med Care* 2005; 43(11): 1130-1139.
