# Supplementary material for: Development and validation of a 30-day mortality index based on pre-existing medical administrative data from 13,323 COVID-19 patients: The Veterans Health Administration COVID-19 (VACO) Index
Source: PLoS One. 2020 Nov 11;15(11):e0241825. doi: 10.1371/journal.pone.0241825 (PMC7657526; doi:10.1371/journal.pone.0241825)
Supplement: S1 File — (DOCX) [file pone.0241825.s003.docx]

**S1 File. VACO Index Calculation of Predicted COVID-19 30-day Mortality**

| **Variable** | **Coefficient** | **Z** | **P-value** | **OR** | **95% CI** |
| --- | --- | --- | --- | --- | --- |
| Age, years |  |  |  |  |  |
| 20-50 | -2.228678713 | -2.83 | 0.005 | 0.11 | 0.02 - 0.50 |
| 50-54 | 0.0 |  | - | 1.00 | - |
| 55-59 | 0.400599289 | 0.97 | 0.334 | 1.49 | 0.66 - 3.36 |
| 60-64 | 0.941322019 | 2.50 | 0.013 | 2.56 | 1.22 - 5.37 |
| 65-69 | 1.295007128 | 3.49 | <0.001 | 3.65 | 1.77 - 7.55 |
| 70-74 | 1.629533438 | 4.55 | <0.001 | 5.10 | 2.53 - 10.3 |
| 75-79 | 1.763345763 | 4.72 | <0.001 | 5.83 | 2.81 - 12.12 |
| 80-89 | 1.927443543 | 4.96 | <0.001 | 6.87 | 3.21 - 14.72 |
| ≥90 | 2.018752269 | 4.39 | <0.001 | 7.53 | 3.06 - 18.54 |
|  |  |  |  |  |  |
| Sex |  |  |  |  |  |
| Female | 0.0 | - | - | 1.00 | - |
| Male | 0.322291449 | 0.88 | 0.377 | 1.38 | 0.68 - 2.82 |
|  |  |  |  |  |  |
| CCI and Age Interaction Term |  |  |  |  |  |
| Age <85 |  |  |  |  |  |
| CCI |  |  |  |  |  |
| 0 | 0.0 | - | - | - | - |
| 1-3 | 0.612122574 | 2.76 | 0.006 | 1.84 | 1.19 - 2.85 |
| 4-5 | 0.825072847 | 3.36 | 0.001 | 2.28 | 1.41 - 3.69 |
| 6-9 | 0.956099733 | 3.84 | <0.001 | 2.60 | 1.60 - 4.24 |
| ≥10 | 1.395164653 | 4.25 | <0.001 | 4.04 | 2.12 - 7.68 |
| Age 85+, any CCI | 1.529325519 | 4.79 | <0.001 | 4.62 | 2.47 - 8.63 |
|  |  |  |  |  |  |
| MI or PVD |  |  |  |  |  |
| No | 0.0 | - | - | 1.00 | - |
| Yes | 0.267265312 | 2.18 | 0.029 | 1.31 | 1.03 - 1.66 |
|  |  |  |  |  |  |
| Constant | -4.216058062 | -8.41 | <0.001 | 0.01 | 0.01 - 0.04 |

Abbreviations: OR = odds ratio; CI = confidence interval; CCI =Charlson comorbidity index; MI = myocardial infarction; PVD = peripheral vascular disease

Calculation of predicted mortality risk:

coefficient_sum_ = Age_coefficient_ + Sex_coeffient_ + CCI_Age_coefficient_ + MI_PVD_coefficient_ + Constant_coefficient_

OR_calc_ = exp(coefficient_sum_)

risk_pred_ = OR_calc_ / (1 + OR_calc_)

Example: 77 year old male with CCI = 5, but no history of MI or PVD

coefficient_sum_ = 1.763345763 + 0.322291449 + 0.825072847 + 0.0 + (-4.216058062) = -1.305348004

OR_calc_ = exp(-1.305348004) = 0. 271078182

risk_pred_ = 0.271078182 / (1 + 0.271078182) = 0.213266333 = 21%
